# Multidetector Computed Tomography Used in Evaluation of Olfactory Fossa Depth in a Tertiary Hospital: A Descriptive Cross-sectional Study

**DOI:** 10.31729/jnma.8578

**Published:** 2024-05-31

**Authors:** Ajay Kumar Yadav, Rajeev Kumar Shah, Neha Yadav, Bipin Koirala, Binit Dev, Sushil Taparia

**Affiliations:** 1 Department of Radiodiagnosis and Interventional Radiology, Birat Medical College & Teaching Hospital, Biratnagar, Morang, Nepal; 2 Department of Otolaryngology, Birat Medical College & Teaching Hospital, Biratnagar, Morang, Nepal; 3 Department of Microbiology & Infectious Disease, Koshi Zonal Hopsital, Biratnagar, Morang, Nepal

**Keywords:** *computed tomography*, *depth*, *ethmoid*, *olfactory fossa*

## Abstract

**Introduction:**

Olfactory fossa (OF) is a depression in most infero-medial portion of anterior cranial fossa formed by cribriform plate, crista galli and lateral lamella of cribriform plate (LLCP). LLCP being thinnest and extremely variable parts, more prone for iatrogenic injury during sinus surgery in case of asymmetric and deep OF. Multidetector computed tomography (MDCT) is frequently used imaging modality in the evaluation of paranasal sinus. The objective of the study is to classify the OF depth according to the Keros classification.

**Methods:**

In this ethically approved prospective, cross-sectional descriptive study, CT scan was done in 530 consecutive patients from February 2022 to July 2023. Coronal CT images of paranasal sinuses and nose were used to measure the OF depth. The data collected was analyzed using SPSS.

**Results:**

Out of 530 patients included in this study, 310 (58.49%) were male and 220 (41.51%) were female with mean age of 40.46±11.56 years. Total of 1060 olfactory fossa were analyzed with mean depth of 4.96±1.88 mm. In our study, 310 (29.24%) had type I, 730 (68.88%) had type II and 20 (1.88%) had type III according to Keros classification.

**Conclusions:**

Keros type II OF is more common. The dangerous type III OF having low prevalence, more commonly seen on right side and in males.

## INTRODUCTION

Functional endoscopic sinus surgery (FESS) is frequently used minimally invasive surgical technique for primary treatment of diseases of paranasal sinus. To avoid complications, anatomical variations of paranasal sinuses including olfactory fossa (OF) using Multidetector computed tomography (MDCT) should be known. Paranasal sinus CT is therefore very valuable and required before FESS. The lateral lamella is thinnest and extremely variable parts of anterior skull base, more prone for iatrogenic injury and its dehiscent is seen in 14%.^1-5^

According to Keros classification, there are three types of olfactory fossa depth and is measured by vertical height of lateral lamella of cribriform plate. The depth of OF in Keros type I is 1-3 mm, 4-7 mm in type II and 8-16 mm in type III respectively. Type III is most dangerous type, associated with higher chance of iatrogenic injury.^3-10^

In Nepal, there are very few literatures available regarding variations of olfactory fossa depth and this study will provide detailed anatomical variations of olfactory fossa depth which will be valuable for the sinus surgery. So this study is carried out with objective to classify the olfactory fossa depth according to the Keros classification.

## METHODS

After institutional ethical committee approval (Ref: IRC-PA-195/2022), this prospective cross sectional descriptive study was done in all patients who attended department of radiology presenting in one of the tertiary care hospitals of eastern region of Nepal from February 2022 to July 2023 fulfilling the inclusion criteria. All patients who underwent contrast enhanced or non-contrast CT scan of brain and paranasal sinuses with age ≥18 years were included in this study. Patients with history of craniomaxillofacial trauma, sinonasal malignancy, previous sinonasal surgery, congenital anomaly and low quality CT images were excluded from the study. All patients underwent noncontrast or contrast enhanced computed tomography (NCCT) of brain and paranasal sinuses (PNS) using 64 slice MDCT scanner (Somatom Perspective, Siemens, Erlangen, Germany) to see the olfactory fossa and ethmoid roof using standard protocol of the institute from base of skull to vertex in supine position using 5 mm slice thickness and 32x0.2 mm collimation with CT parameters of 120 kVp tube voltage, 210 effective mAs, slice thickness 5 mm, pitch 1.2, field of view 15 cm and rotation time 0.5 s. Reconstruction of images were done in all planes in 1 mm of thin section with 0.5 mm interval.

A single radiologist having more than ten years of experience measured the olfactory fossa depth on coronal CT images of paranasal sinuses on both sides to avoid observer bias using a dedicated CT work station in bone widow.^7,9^ The olfactory fossa depth was measured after identifying following anatomical structures and point on coronal CT images like cribriform plate (CP), lateral lamella of the cribriform plate (LLCP) and medial point of ethmoidal roof (medial end of ethmoid roof articulating with LLCP). Horizontal lines were drawn along the cribriform plate and at the medial ethmoid roof point. The vertical height of lateral lamella was measured between these two horizontal lines using the distance measuring tool. The vertical height of lateral lamella corresponds to the depth of OF. The heights of both right and left lateral lamella were recorded separately. The olfactory fossa depth was classified according Keros classification as type I (1 to 3 mm depth), type II (4 to 7 mm depth) or type 3 (more than 7 mm depth). Asymmetry in the depth (difference of more than 3 mm) between the right and left olfactory fossa was also recorded. All the findings were noted in specifically designed proforma and entered in MS excel for further analysis.

The collected data was symmetrically distributed. Data was analyzed using SPSS software and results were expressed in mean±standard deviation for continuous data and median for noncontinuous data. Results were also presented in Tables where necessary.

## RESULTS

Out of 530 patients, 310 patients (58.49%) were male while 220 patients (41.51%) were female with mean age of 40.46±11.56 years (age range between 18-85 years).

Among the 530 patients, a total of 1060 olfactory fossa were analyzed with mean depth of 4.96±1.88 mm with range from 1 mm to 14.5 mm. The mean depth of OF on right side was 5.02±1.89 mm while mean depth of OF on left side was 4.89±1.87 mm. The mean OF depth in males was 5.03±1.92 mm while mean depth of OF in females was 4.75±1.79 mm.

Among the 530 patients with total of 1060 sides, Keros type II was seen in 730 (68.88%) sides followed Keros type I which was seen in 310 (29.24%) sides and the least common Keros type III was seen in 20 (1.88%) sides. On the right side, Keros type I was seen in 160 (30.18%) olfactory fossa (Table 1).

**Table 1:** Distribution of olfactory fossa according to the side and Keros classification (n= 1060).

Keros type	Right	Left	Total
Type I	160 (30.18%)	150 (28.30%)	310 (29.24%)
Type II	358 (67.54%)	372 (70.18%)	730 (68.88%)
Type III	12 (2.28%)	8 (1.52%)	20 (1.88%)
Total	530 (100%)	530 (100%)	1060 (100%)

Among the males, Keros type I olfactory fossa was seen in 88 (28.38%) sides, Keros type II was seen in 214 (69.03%) sides and Keros type III was seen in 8 (2.59%) sides on the right side. Among the females, Keros type I OF was seen in 72 (32.72%) sides, Keros type II was seen in 146 (66.36%) sides and Keros type III was seen in 2 (0.92%) sides on the right side. Among the males, Keros type I olfactory fossa was seen in 82 (26.45%) sides, Keros type II was seen in 222 (71.61%) sides and Keros type III was seen in 6 (1.94%) sides on the left side. Among the females, Keros type I OF was seen in 68 (30.90%) sides, Keros type II was seen in 148 (67.27%) sides and Keros type III was seen in 4 (1.83%) sides on the left side (Table 2).

**Table 2:** Showing distribution of olfactory fossa based on Keros classification according to their sides and gender (n= 1060).

Keros type	Right/M	Right/F	Left/M	Left/F	Total/M	Total/F	Overall
Type I	88 (28.38%)	72 (32.72%)	82 (26.45%)	68 (30.90%)	170 (27.41%)	140 (31.81%)	310 (29.24%)
Type II	214 (69.03%)	146 (66.36%)	222 (71.61%)	148 (67.27%)	436 (70.32%)	294 (66.81%)	730 (68.88%)
Type III	8 (2.59%)	2 (0.92%)	6 (1.94%)	4 (1.83%)	14 (2.27%)	6 (1.38%)	20 (1.88%)
Total	310 (100%)	220 (100%)	310 (100%)	220 (100%)	620 (100%)	440 (100%)	1060 (100%)

M- Male, F- Female

The olfactory fossa was deeper on right side in males with right side was deeper in 170 (54.84%) and left side was deeper in 131 (42.25%). In females, the olfactory fossa was deeper on left side with left side was deeper in 115 (52.27%) and right side was deeper in 97 (44.09%). Overall right side was deeper and seen in 267 (50.37%) cases. Symmetrical OF was seen in 17 (3.20%) cases while asymmetrical OF was seen in 513 (96.80%) cases. Asymmetry was taken when difference between depth of OF on both right & left sides was more than 0.1 mm.

## DISCUSSION

With the advancement in multidetector computed tomography (MDCT) using thin CT images of paranasal sinuses and nose (mainly in coronal plane) provides early diagnosis with detection of disease process and the treatment of the diseases besides providing good anatomical details of olfactory fossa and base of skull. In the present time, functional endoscopic sinus surgery is treatment of choice for paranasal sinuses disease for clearance of disease and improving good ventilation of paranasal sinuses. During the surgery, there is chance of iatrogenic injury to the olfactory fossa and basal of skull with the thinnest bone LLCP is most commonly vulnerable to injury. So CT scan of paranasal sinuses in coronal images provides good anatomical delineation of base of skull and olfactory fossa besides anatomical variations of them. In 1962, Keros P published study of olfactory fossa in cadaver and described a classification based on the study which was used in many published literatures in the present date.^11-20^

In our study, the mean age of the patients was 40.46±11.56 years and majority of them were male (58.49%). Most of the patients showed Keros type II OF. Similar findings were seen in studies done by Babu et al8, Sarloo et al^2^, Naidu et al,^11^ and Patil et al,^9^ where mean age of the patients were 44.5, 36.8, 37.6 and 44.2 years respectively and majority of them were males.

In our study, Keros type II OF was the most common type seen in 68.9% followed by type I OF seen in 29.24% and the least common is type III OF seen in 1.88%. The surgery is safe in type I and type II OF while it is dangerous in type III OF. Most of the published studies worldwide also showed Keros type II OF is the most common type followed by type I OF and type III OF similar to our study. The results of our study were similar to the original study done by Keros in Germany in 1962 showed type II OF being the most common type followed by type I and the least common type being type III OF. Some studies also showed Keros type I OF is the most common type followed by type II OF and the least common type being type III OF. In Nepal, there were two published studies showing Keros type I OF being the most common type followed by type II and type III and the results were not similar to our study. There is wide variation in depth and types of olfactory fossa based on Keros classification worldwide due to different racial population, ethnicity and regions. This results also suggest that there is chance of intracranial injury in these populations during FESS surgery through lateral lamella. Majority of the studies suggested that objective assessment of anatomy and fine details of olfactory fossa and anterior skull base provided by Keros classifications help surgeons during operative procedures and to take necessary safety routes and plan of the surgery to avoid complications and iatrogenic intracranial penetrations.

A comparison of the Keros classification among different studies reveals notable variations in the distribution of Type I, II, and III classifications across countries. In studies conducted in India, such as those by Babu et al. (Type I: 17.5%, Type II: 74.6%, Type III: 7.9%),^8^ Salroo et al. (Type I: 29%, Type II: 61%, Type III: 10%),^2^ Pawar et al. (Type I: 18.5%, Type II: 74.5%, Type III: 7%),^12^ Satish Nair (Type I: 17.2%, Type II: 77.2%, Type III: 5.6%),^15^ and Patil et al. (Type I: 21.6%, Type II: 72.8%, Type III: 5.6%),^9^ Type II predominates, though with varying degrees. Studies from other countries also present different distribution patterns. For instance, research from Germany (Keros P3: Type I: 12%, Type II: 70%, Type III: 18%),^3^ the USA (Solares et al.: Type I: 83%, Type II: 15%, Type III: 2%),^13^ and the UK (Gauba et al.: Type I: 34.4%, Type II: 28.1%, Type III: 37.5%),^14^ shows diverse distributions. Similarly, studies from the Philippines (Justin Elfred et al.: Type I: 81%, Type II: 17.9%, Type III: 0.5%),^5^ and (Paber et al.: Type I: 81.8%, Type II: 17.7%, Type III: 0.5%),^4^ exhibit a significant prevalence of Type I and very low percentages of Type III. Notably, studies from Nepal, including Bista et al. (Type I: 86%, Type II: 12%, Type III: 2%),^20^ Shrestha BL et al. (Type I: 86.1%, Type II: 13.4%, Type III: 0.5%),^7^ and our study (Type I: 29.2%, Type II: 68.9%, Type III: 1.89%), indicate a high prevalence of Type I classifications, with our study showing a particularly high proportion of Type II classifications. These findings underscore the variability in Keros classification across populations and regions.

Type III OF was seen in 1.9% patients in our study which is dangerous type OF. Keros type III was seen more on right side (2.3%) than on left side (1.5%), more in males (2.3%) than in females (1.4%) and also more on right side even in males (2.5%) shown in table 1 and 2.

In our study, the mean depth of olfactory fossa was 4.96mm. The mean depth of olfactory fossa in studies by Babu et al^8^, Sarloo et al^2^, Patil et al^9^, Naidu et al^11^, Almushayti ZA et al10 and Shrestha BL et al^7^ was 5.26 mm, 5.08 mm, 5.3 mm, 5.75 mm, 5.1 mm and 3.1 mm respectively. In our study, Keros type II olfactory fossa was the most common type olfactory fossa within the both genders and also on the both right and left sides. Deeper OF fossa seen in males in comparison to females in our study with mean OF depth in males was 5.03±1.92 mm while mean depth of OF in females was 4.75±1.79 mm. Similar findings with deeper OF in males was than females were seen in studies by Babu et al8, Naidu et al^11^ and Sarloo et al.^2^

In our study, deeper olfactory fossa was seen on right side in males (54.8%) while in females, the olfactory fossa was deeper on left side (52.3%) however overall right side was deeper and seen in 50.4% cases. Symmetrical OF was seen in 3.2% cases while asymmetrical OF was seen in 96.8% cases. Similar results were seen in study done by Babu et al8 where symmetrical OF was seen in 25% and asymmetrical OF was seen in 75% cases. Due to more dedicated measurement of OF depth and the more accurate definition of asymmetry (difference >0.1 mm) compared with other studies, there is higher rate of asymmetry seen in these studies. The majority of the participants had difference of ≤1 mm and very few had >2 mm difference between two sides in our study. Asymmetry was seen in 11.7% and 14.6% of patients in the study done by Satish Nair et al and Ali et al.

In our study, the dangerous Keros type III is the least common type and seen more in males than in females with more on right side. Because of the long length of the lateral lamella in Keros type III olfactory fossa, it is more vulnerable to itrogenic injury during frontoethmoidal surgery.

With the advancement in technology of computed tomography, it provides great information and helped a lot in the field of medicine for the diagnosis, for evaluation of sinonasal diseases and providing precise anatomical details with variations. So MDCT should be included in preoperative evaluation of the diseases of paranasal sinuses and nose so that the operating surgeon's should have good anatomical knowledge of olfactory fossa and base of skull for further planning of safe surgical procedures. To avoid complications during surgical procedures, anatomical variations with asymmetry of olfactory fossa and adjacent structures should be kept in mind.

This was a single institutional study and done in one region of the country with shorter duration of the study. The measurements were taken by single radiologist. The study was done in patients above the age 18 years with exclusion of paediatric populations.

In our study, Keros type II OF is most common type followed by Keros type OF and overall OF is deeper on right side. However this is a single institutional study and done in eastern region of Nepal so we recommend a larger multi-institutional study should be done in different regions of the country in paediatric and adults patients to generalize the Keros classification and provides more detailed information.

## CONCLUSIONS

Keros Type II olfactory fossa were most common type followed by Type I and Type III. The dangerous Keros type III being least common type seen more commonly in males and more on right side susceptible for iatrogenic injury, thus detailed knowledge of anatomy of olfactory fossa is mandatory according to Keros classification before planning surgery in safer way. In patients with diseases of paranasal sinuses and nose, preoperative wok up of olfactory fossa with MDCT evaluation to get adequate and fine anatomical details with anatomical variations of the olfactory area and anterior base of skull is needed to avoid damage to lateral lamella of the cribriform plate and adjacent structures of the olfactory analyzer to prevent iatrogenic intracranial penetrations along with complications like rhinoliquorrhoea, meningitis, loss of the sense of smell, and brain abscesses.
